# Stem cells in the treatment of central nervous system disease

**DOI:** 10.1007/s00415-018-8818-7

**Published:** 2018-03-09

**Authors:** Duncan McLauchlan, Neil P. Robertson

**Affiliations:** Department of Neurology, Institute of Psychological Medicine and Clinical Neurosciences, Cardiff University, Cardiff, CF14 4XW UK

Stem cells are dividing cells that exhibit potency (ability to subdivide into multiple different cells types) and self-renewal (ability to undergo multiple cell cycles without differentiation). The degree of potency can be considered as totipotent (can divide into any cell type), pluripotent (descendants of totipotent cells and can divide into cells from endoderm, mesoderm, or ectoderm), or multipotent (division is limited to cell types from a single family). Stem cells are found in embryos, cord blood, and (in adults) bone marrow and stromal tissue. In 2006, Yamanaka demonstrated that adult stem cells may be induced to form pluripotent stem cells, i.e., could increase their potency. This and other advances in stem cell research have generated increasing interest across a broad range of medical specialities because of the potential for therapeutic effects such as replacement of damaged cells, repair of damaged tissue by merging with endogenous cells, paracrine functions, and immunomodulatory effects.

The central nervous system (CNS) undergoes little endogenous neural cell division beyond selected regions such as the hippocampus and striatum. This limits the capacity of the brain and spinal cord to undergo repair and therefore results in more severe consequences from tissue damage. In addition, the lack of endogenous repair mechanisms almost certainly contributes to the widespread failure of many potential therapeutic agents hitherto trialed in degenerative CNS diseases, and the consequent paucity of effective disease modifying drugs currently available. In treating disease, therapeutic approaches can employ a wide range of targets, but, broadly, these fall into either a cessation of a pathological process, an enhancement of a protective mechanism, or regeneration of damaged tissue. The application of stem cell therapeutics in CNS disease has the potential to address all three pathways and generated understandable excitement in the neuroscience community. However, as with all research endeavours, enthusiasm should be tempered with robust biological rationale, rigorous ethical and methodological oversight, and clear and consistent trial methodology to show the evidence of benefit.

This month’s journal club covers three papers dealing with stem cells as therapeutic interventions in CNS disease. First, a randomised-controlled trial of intravenous mesenchymal stem cells as an immunomodulatory therapy to reduce inflammatory mediated neural damage in stroke; second, a dopaminergic cell replacement in an animal model of Parkinson’s disease. Finally in a break from our usual convention for journal club; an important overview and guidance for research using stem cells for immunomodulation and brain repair in multiple sclerosis.

## Safety and efficacy of multipotent adult progenitor cells in acute ischaemic stroke (MASTERS): a randomised, double-blind, placebo-controlled, phase 2 trial

To date, there are no effective treatments for neuroprotection or brain repair in stroke. The previous studies of stem cell treatment in stroke have been small, single-centre studies. This is the first large multi-centre study of intravenous multipotent stem cells in the treatment of cerebral damage secondary to stroke.

MASTERS was a phase II randomised-controlled trial, with primary outcomes for safety and efficacy. Patients with a confirmed anterior circulation infarction on MRI treated with thrombolysis, aged 18–79, and with an NIHSS score of 8–20 were recruited. Cells were given intravenously between 24 and 48 h after the index event. The initial 8 patients received 400 million cells/kg of body weight; a planned dose escalation increased this to 1200 million cells/kg. Participants were randomised by computer, the intervention group received cells plus vehicle, and the control group received vehicle alone. Participants were assessed at 7, 30, 90, and 365 days using the modified Rankin Scale (mRS), NIHSS, and Barthel index scales; exploratory outcomes were MR volume change and levels of cytokines and regulatory T cells. Primary safety outcomes were dose-limiting toxic events at 7 days after infusion, allergic/adverse events secondary to cells, or worsening NIHSS of 4 points. Secondary safety outcomes were diagnosis of infection, mortality, or changing vital signs. The primary efficacy outcome was a compound outcome of mRS < 2, NIHSS improvement of > 75%, and a Barthel score of > 94. Secondary outcomes were change in mRS, improvement in NIHSS to < 1 or by more than 10 points, and ‘excellent outcome’ (Barthel > 94, mRS 0–1, NIHSS 0–1). Data were analysed on an intention to treat basis.

129 patients were randomised, 8 patients received the lower dose of cells, 65 patients received the cells, and 61, placebo. The groups were well matched for age and median NIHSS score. There were no differences on any of the safety outcomes. There were also no differences on any of the primary efficacy outcome measures. An improvement on only one of the secondary outcomes was seen: “excellent outcome”.

*Comments and conclusions.* There were no differences between the groups on any of the safety outcomes. Although this trial was not powered to look for efficacy outcomes, one of the secondary outcomes did show an improvement in the intervention group; however, the significance level would not have been met after correction for the family-wise error rate. Furthermore, the biological rationale for intravenous mesenchymal stem cells in stroke is not compelling based on the cited animal literature: the authors hypothesise that stem cells may act as immunomodulators, rather than replace damaged neurons and glia.

Hess DC et al. (2017) Lancet Neurology 16:360–368.

## Human iPS cell-derived dopaminergic neurons function in a primate Parkinson’s disease model

Previous work has demonstrated that it is possible to successfully graft primates with nigro-striatal lesions, with neurons derived from embryonic stem cells and human fetal stem cells. This is the first study demonstrating that it is possible to graft these primates with induced Pluripotent Stem (iPS) cells derived from humans.

Cells were derived from 3 patients with Parkinson’s disease (screened for common genetic Parkinson’s mutations) and 5 healthy controls. IPS cells were induced using standard methods. Cells were stained for floor-plate markers and patch-clamp stimulation to confirm successful differentiation and function as midbrain neurons. 11 MPTP lesioned cynomolgus monkeys were used in the experiment. They were split into 3 groups and had putaminal grafts: either iPS cells from PD patients, iPS cells from controls, or vehicle only (i.e., no cells). Graft growth, tumorogenesis, and function were measured using MRI and PET scans. The monkey’s movement disorder was assessed by blinded observers using a standardised assessment, and also by pixel change on standardised videos. The animals were then euthanised and tumorigenic markers were assessed histologically. ANOVAs were used for group comparison, and linear regression for change over time.

Scores on both the formalised examination and video-analysis were significantly higher in the grafted groups than the ungrafted groups. MR analysis showed an increase in graft size (mean peak volume 39.4 mm^3^) until 6–9 months and a plateau of volume thereafter. PET analysis showed no tracer uptake of FLT (a marker of tumorogenesis); survival and function of the dopaminergic graft were confirmed in all grafted animals. There were no differences on any of the measures between grafts from PD patients and normal controls. No monkey had evidence of inflammatory infiltrate or rosette-forming cells to suggest tumour formation or host-mediated attack on the graft.

*Comments and conclusions*. We consider this to be a study of considerable importance; it is the first demonstration that human-derived iPS stem cells can form dopaminergic neurons, be safely grafted, and restore function in animals with an objective movement disorder. The fact that there was no difference in cell lines derived from PD patients and human controls, suggests that PD patients could form their own transplants obviating the need for allograft and immunosuppression. The sample size is small, and the method for choosing which animals to graft is unclear. Nevertheless, this study offers encouragement in using this methodology for the potential future treatment of Parkinson’s disease.

Kikuchi T et al. (2017) Nature 548:592–609.

## Cell-based therapeutic strategies for multiple sclerosis

A wide variety of approaches and cell lines have been employed to interrogate the potential for stem cell therapies in multiple sclerosis (MS). Current disease modifying therapies (DMTs) carry significant side effects, and there are no current effective treatments for progressive disease or cell repair. This review article summarises the current evidence base in this field and offers guidelines for future research and clinical practice.

Immunoablation and Autologous Haematopoietic Stem Cell Transplant (IAHSCT): The rationale for this approach is to modify the immune system by ablation and replacement of the host-sensitised immune system. Evidence has accrued that this approach switches the immune response to a Th1-mediated pathway, and autoreactive T cells are reduced. Initially this methodology was employed in progressive patients with little success; however, more recently, it has been used for highly aggressive relapsing–remitting disease with more promising results. Researchers have used an ablative agent (cyclophosphamide and alemtuzumab) followed by GCSF to re populate the immune system. Early approaches used busulphan and whole-body irradiation which resulted in unacceptable toxicity. There are few head-to-head trials with conventional DMTs, but retrospective analysis of the published data suggests that NEDA (no evidence of disease activity) is achieved in up to 80%, compared with 46% on conventional DMTs. Mortality is less than 5%, with serious adverse events in 9%. The authors recommend (1) selecting relapsing–remitting patients with highly active disease, who have failed on DMTs, (2) head-to-head trials with conventional DMTs; and (3) any patients treated outside a clinical trial should be entered on a register.

*Mesenchymal stem cells*. Mesenchymal stem cells (MSCs) are present in almost all body tissues; they are involved in tissue repair and also have a paracrine function; which may mediate their effects in the CNS. There are a number of candidate mechanisms for their effects: re-myelination, angiogenesis, neuroprotection, and reduced gliosis. However, there remain many uncertainties: the optimal dose of cells, whether the purification of the cell line is necessary, and, finally, the need for pre-differentiation into neural progenitor cells. The lack of agreed end-points or outcomes for clinical trials is a major sticking point for future research, although, as most studies are at phase II, safety outcomes (such as tumour growth) are paramount. Most MSCs are delivered intravenously, and there is some evidence to suggest they do reach the CNS, although some workers have delivered them directly into the CSF. There have been a few adverse events—albeit including 1 CNS malignancy. Two randomised-controlled trials have been negative. The authors highlight the need for standardisation of cell dose, number of infusions, and clarification of the need for purification of the cell lines prior to transplant.

Oligodendrocyte progenitor cells (OPCs) and induced pluripotent cells (iPCs): Animal work has shown widespread re-myelination in a mouse model of MS treated with OPCs, the most myelinogenic cell lines can be selected by testing for PDGF alpha and CD140a. These cells must be injected into the CNS to act, but, by what mechanism, they localise to tissue damage (or if they do) remains unclear. Fetal cells are the main source of OPC, which necessitates immunosuppression. IPSCs obviate the need for immunosuppression but may have higher neoplastic potential than OPCs. A phase 1 trial is underway.

*Ethical issues and future directions*. The authors recommend that all patients are informed of the experimental nature of these treatments, and research should adhere to relevant national and international guidelines. They further recommend an international registry for patients undergoing this treatment.

*Comments and conclusions.* IAHSCT has the most evidence to support its use in MS; however, it is unclear whether observed efficacy occurs as a consequence of aggressive immunosuppression, or alteration of the immune response. Evidence to support the use of MSCs in MS is sparser; and there is a lack of agreement in the literature about delivery method, dose, cell lines, and even mechanism of action. OPCs and iPSCs have only shown promise in animal models to date.

Scolding J et al. (2017) Brain 140:2776–2796.

